# Precision Dosing of Meropenem in Adults with Normal Renal Function: Insights from a Population Pharmacokinetic and Monte Carlo Simulation Study

**DOI:** 10.3390/antibiotics13090849

**Published:** 2024-09-05

**Authors:** Yong Kyun Kim, Gaeun Kang, Dae Young Zang, Dong Hwan Lee

**Affiliations:** 1 Division of Infectious Diseases, Department of Internal Medicine, Hallym University Sacred Heart Hospital, Hallym University College of Medicine, Anyang 14066, Republic of Korea; amoureuxyk@hallym.or.kr; 2 Division of Clinical Pharmacology, Chonnam National University Hospital, Gfwangju 61469, Republic of Korea; bp00092@cnuh.com; 3 Division of Hematology-Oncology, Department of Internal Medicine, Hallym University Sacred Heart Hospital, Hallym University College of Medicine, Anyang 14066, Republic of Korea; fhdzang@hallym.or.kr; 4 Department of Clinical Pharmacology, Hallym University Sacred Heart Hospital, Hallym University College of Medicine, Anyang 14066, Republic of Korea

**Keywords:** meropenem, population pharmacokinetics, noncompartmental analysis, Monte Carlo simulation, normal renal function, healthy, adult

## Abstract

This study aimed to develop a population pharmacokinetic (PK) model for meropenem in healthy adults and explore optimal dosing regimens for patients with normal renal function. PK samples were obtained from 12 healthy participants, which were analyzed using noncompartmental analysis and nonlinear mixed-effect modeling. The PK profiles of meropenem were characterized using a two-compartment model, and serum creatinine level was identified as a significant covariate affecting total clearance. Monte Carlo simulations were conducted using this model to inform dosing recommendations. The target index for meropenem efficacy was defined as the cumulative percentage over 24 h during which free (*f*) drug concentration exceeded the minimum inhibitory concentration (MIC) under steady state conditions (*f*T_>MIC_). These simulations indicated that the current dosage regimen of 1 g for 30 min infusions every 8 h achieved a 90% probability of target attainment (PTA) for 40%*f*T_>MIC_ when the MIC was <2 mg/L. However, to achieve more stringent therapeutic targets, such as a 90%PTA for 100%*f*T_>MIC_ or a 90%PTA for 100%*f*T_>4MIC_, higher doses administered as 3 h extended infusions or as continuous infusions may be necessary. These results highlight the need for model-informed precision dosing to enhance the efficacy of meropenem therapy across various MIC levels and therapeutic targets.

## 1. Introduction

Meropenem belongs to the carbapenem class of β-lactam antibiotics and is distinguished by its activity against a broad spectrum of Gram-positive and Gram-negative bacteria [1,2]. This broad-spectrum activity is important for managing complex infections in intensive care units (ICUs), particularly those caused by multidrug-resistant organisms, which is a challenge in these high-risk settings [3]. Carbapenems disrupt bacterial growth by interacting with penicillin-binding proteins, which are required for cell wall synthesis. Their structure confers resistance to degradation by β-lactamase enzymes, which is a common bacterial resistance mechanism [4]. Because of its resistance to hydrolysis, carbapenems are particularly valuable for treating severe hospital-acquired infections, including those from extended-spectrum beta-lactamase-producing *Enterobacterales* [3,4].

Although carbapenems are highly effective, their increased use is associated with an increase in resistance among Gram-negative bacteria to these drugs, which highlights the urgent need to administer them prudently to prevent further resistance. Carbapenems rank as the third most frequently administered antibiotics worldwide for community-acquired infections in ICUs. This represents 10.7% of such treatments, and they are the preferred antibiotic for managing nosocomial infections, accounting for 21.5% of all such cases [5]. This significant reliance on carbapenems emphasizes the need for continued efforts to refine their use and ensure their effectiveness against evolving drug-resistant bacteria. The emerging problem of antimicrobial resistance, which is considered a major global public health threat, contributed to approximately 1.27 million fatalities worldwide in 2019 [6]. This issue highlights the importance of optimizing antimicrobial dosing to avoid inadequate treatment outcomes and the development of drug resistance. The concept of “mutant prevention concentration” is fundamental in this context, as insufficient antimicrobial levels enable resistant bacterial populations to flourish [7].

Given the pivotal role of meropenem in the treatment of severe infections, it is critical to understand meropenem’s behavior in different patient populations. Meropenem is primarily excreted unaltered by the kidneys. Studies focusing on precision dosing of meropenem are often done in critically ill patients, who typically exhibit impaired renal function. Consequently, these findings may not be directly applicable to patients with normal or enhanced renal function. Some studies suggest that meropenem dosage should be increased for cases in which renal function is normal or enhanced [8,9,10]. The purpose of the present study was to analyze the population pharmacokinetic (PK) characteristics of meropenem in healthy adults. The data obtained from individuals without confounding factors, such as illness, not only provide standard PK parameters, but also contribute to the development of predictive models for precision dosing when used for subsequent patient-focused research. Another aim of this study was to use this population PK model to conduct Monte Carlo simulations and establish optimal dosing regimens for patients with normal renal function.

## 2. Results

### 2.1. Participants

Informed consent was obtained from 14 healthy volunteers; however, two individuals were excluded because of positive results from allergic skin tests. The demographic and clinical characteristics of the remaining participants (four females and eight males) are listed in Table 1. The laboratory characteristics displayed clinically insignificant values. Creatinine clearance (CL_CR_), which was calculated using the Cockcroft–Gault formula, exhibited values comparable to those estimated using the CKD-EPI formulas for glomerular filtration rate (eGFR). A body surface area (BSA) of 1.73 m^2^ was used to standardize the CL_CR_. The eGFR values were adjusted for BSA levels in each patient. No adverse events were reported among the participants administered meropenem.

### 2.2. Population Pharmacokinetic Analysis

Plasma samples (n = 84) were analyzed to characterize the PK profile of meropenem, which was best described by a two-compartment model. The objective function values (OFVs) for the one-, two-, and three-compartment base models were −43.231, −150.154, and −160.715, respectively. Although the three-compartment model exhibited lower OFVs, it was not selected as the structural model because the clearance was unacceptably low at 0.0138 L/h, and the volume of distribution for the second peripheral compartment was excessively high at 4404 L. In addition, the RSEs for all parameters could not be estimated. Therefore, the two-compartment model was considered the most appropriate structural base model (Table S1). The two-compartment model incorporated total clearance (CL), volume of distribution in the central compartment (V1), volume of distribution for the peripheral compartments (V2), and intercompartmental clearance between V1 and V2 (Q2), as listed in Table 2. Because of the high correlation (0.99) between the variabilities (ω^2^) of CL and V1, these parameters were coded to share a single η, which was formulated as follows: CL = TVCL × EXP[ETA(1)], and V1 = TVV1 × EXP[THETA(1) × ETA(1)], where TV represents the typical value. THETA(1) was estimated to be 1.59 in the base model (Table S1) and 1.53 in the final model (Table 2). The stepwise covariate selection process is detailed in Table S2. The final PK model, which achieved an OFV of −175.610, identified serum creatinine levels as a significant factor that influences CL (Table 2).

**Figure 1:**
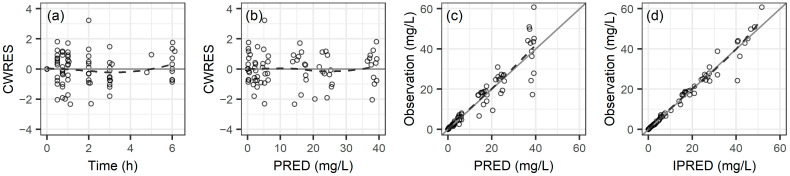
Goodness-of-fit plots for the final pharmacokinetic model of meropenem: (**a**) conditional weighted residuals (CWRES) vs. time, (**b**) CWRES vs. population predicted concentration (PRED), (**c**) observed concentration vs. PRED, and (**d**) observed concentration vs. individual predicted concentration (IPRED). Smooth curves are shown by the dashed lines.

Figure 1 shows the goodness-of-fit plots for the final PK model. It also shows that the distribution of conditional weighted residuals and observed concentrations closely align with the x-axis and the unity line. This indicates minimal bias in the PK parameters and affirms the appropriateness of the model. The individual fit plots for meropenem are provided in Figure S1. Figure S2 shows the visual predictive checks (VPCs) for the meropenem PK model in which the 10th, 50th, and 90th quantiles of the observed concentrations mostly fall within the 95% confidence interval of the simulated data. This underscores the model’s robust predictive accuracy and reliable depiction of the observed concentrations.

### 2.3. Comparing Noncompartmental Analysis and Population Pharmacokinetics Results

Table 3 lists the findings for the descriptive statistical analysis of the PK parameters for each subject, which were derived from the NCA and population PK analysis. Following the intravenous administration of 500 mg of meropenem, the average (coefficient of variation, CV%) was 40.2 (30.1%) mg/L for C_max_ and 40.4 (22.7%) mg·h/L for AUCinf. Comparing the results analyzed using NCA and population PK through the Wilcoxon signed-rank test, the half-lives t_1/2λz_ and t_1/2β_ were not statistically different (*p* = 0.0712). However, the CL_NCA_ and CL and the V_ssNCA_ and V_ss_ showed statistically significant differences (*p* = 0.048 and 0.002, respectively).

### 2.4. Dosage Simulation

**Figure 2:**
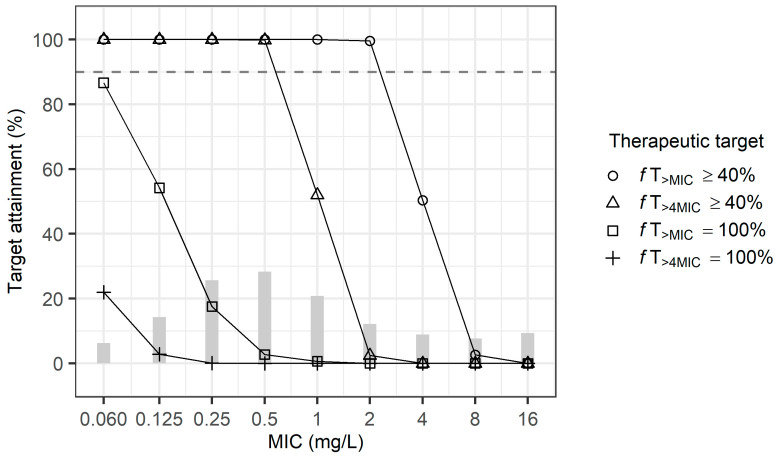
Probability of target attainment for empirical meropenem therapy (1 g q8h) in patients with normal renal function, which highlights MIC distribution for *P. aeruginosa*.

Figure 2 shows the probability of target attainment (PTA) for the empirical therapy using the current dosage regimen (1 g every 8 h) in patients with normal renal function as determined in the first simulation. This reflects the distribution of meropenem MICs for *Pseudomonas aeruginosa* as reported by the European Committee on Antimicrobial Susceptibility Testing (EUCAST) in 2024 [11]. The target index for meropenem was defined as the cumulative percentage over 24 h during which the free (f) drug concentration exceeded the minimum inhibitory concentration (MIC) under steady state conditions (*f*T_>MIC_). The regimen achieved a 90%PTA for 40%*f*T_>MIC_ when the MIC was below 2 mg/L; however, it failed to reach a 90%PTA for 40%*f*T_>4MIC_ when the MIC exceeded 1 mg/L. In addition, when the targets were set at 100%*f*T_>MIC_ or 100%*f*T_>4MIC_, the current regimen did not achieve a 90%PTA at any MIC level.

**Figure 3:**
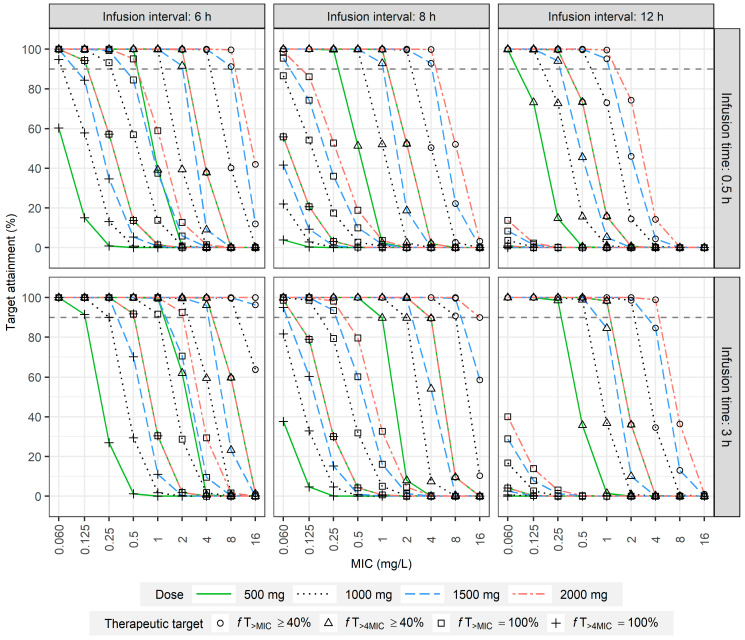
PTA for intermittent infusion of meropenem in adults with normal renal function. The analysis was performed at four dose levels (500, 1000, 1500, and 2000 mg) and three dosing intervals (6, 8, and 12 h).

In the second simulation, the optimal dosage regimens were evaluated to achieve a PTA ≥ 90% at 40%*f*T_>MIC_, 40%*f*T_>4MIC_, 100%*f*T_>MIC_, and 100%*f*T_>4MIC_ (Figure 3). Because 2 g is four times the dose of 0.5 g, the PTA for *f*T_>4MIC_ at a dose of 2 g overlapped with the PTA for *f*T_>MIC_ at a dose of 0.5 g. When 1 g of meropenem was infused over 0.5 h every 12 h, the PTA for 40%*f*T_>MIC_ exceeded 90% when the MIC was at or below 0.5 mg/L. Extending the infusion time to 3 h resulted in a PTA > 90% when the MIC was 2 mg/L and >80% when the MIC was 4 mg/L. For a 1.5 g dose of meropenem infused over 0.5 h every 8 h, the PTA for 40%*f*T_>4MIC_ exceeded 90% when the MIC was at or below 1 mg/L. Increasing the infusion duration to 3 h enabled the PTA to exceed 90%, even when the MIC was 2 mg/L. When 2 g of meropenem was infused over 0.5 h every 6 h, the PTA for 100%*f*T_>MIC_ exceeded 90% when the MIC was at or below 0.5 mg/L. Extending the infusion time to 3 h maintained a PTA > 90%, even at an MIC of 2 mg/L; however, even with a 3 h infusion interval of 2 g meropenem every 6 h, the PTA for 100%*f*T_>4MIC_ did not exceed 90% when the MIC was above 0.5 mg/L.

**Figure 4:**
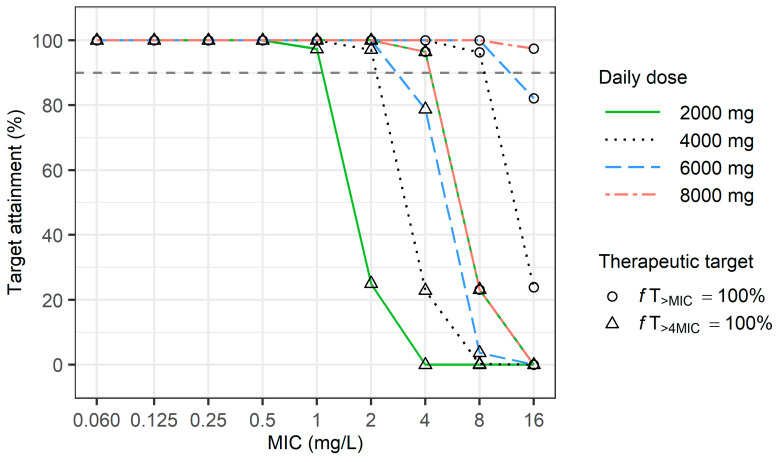
PTA for continuous meropenem infusions in adults with normal renal function at daily meropenem doses ranging from 2000 to 8000 mg.

In the final simulation, the optimal dose regimens for continuous infusion were evaluated to achieve a PTA ≥ 90% for both 100%fT_>MIC_ and 100%fT_>4MIC_ (Figure 4). When 2 g of meropenem was administered as a continuous infusion, the PTA for 100%*f*t_>4MIC_ exceeded 90% when the MIC was 1 mg/L, and the PTA for 100% *f*t_>MIC_ also exceeded 90% when the MIC was 4 mg/L. When 8 g of meropenem was administered as a continuous infusion, the PTA for 100%*f*t_>4MIC_ exceeded 90% when the MIC was 4 mg/L.

## 3. Discussion

The primary objective in the realm of medical treatment is to restore patients from a diseased to a healthy state. Although some conditions may be irreversible or chronic, others are temporary or reversible, such as renal function. Research on a model-informed precision dosing of antibiotics often focuses on patients with decreased or severely impaired renal function, which sometimes overlooks those with enhanced renal activity. This can result in a form of reverse discrimination in which individuals with normal renal function are inadvertently neglected. This is more likely to be the case with drugs that have been in use for a long time. The rationale for initiating clinical trials with healthy individuals during the drug development process is the ability to establish the effects of the body on a drug and of a drug on the body, without the confounding factors that may be present in patients. This standard data may serve as a benchmark for clinical trials in patients. The methodologies used in developing PK models for precision dosing should also reflect this approach. Models developed exclusively for specific patient groups often show limited predictability. The present study was conducted to establish a model for meropenem with excellent predictive ability using PK data from healthy individuals. Although the ultimate goal was to integrate this model with patient data to enhance therapeutic outcomes, the findings from healthy adults alone have significant value. To date, there has been no population PK study conducted on healthy Korean individuals for meropenem, which indicates the uniqueness and importance of this endeavor.

In the present study, the PK profiles of meropenem were characterized using a two-compartment model in which serum creatinine was a significant covariate affecting clearance (CL). Specifically, CL decreased from 14.3 L/h at a serum creatinine level of 0.6 mg/dL to 11.7 L/h at 1.0 mg/dL. The population PK analysis exhibited typical values for total clearance and the steady state volume of distribution (Vss, V1 + V2) of 12.5 L/h and 12.32 L, respectively (Table 2). The NCA indicated that the mean values of CL_NCA_ and V_ssNCA_, based on a body weight of 70 kg, were 14.07 L/h and 15.44 L, respectively. Moreover, the mean post-hoc estimates for CL and V_ss_ from the population PK analysis, which were also based on a body weight of 70 kg, were 13.79 L/h and 13.79 L, respectively (Table 3). These findings are comparable to those of PK studies conducted in healthy adults (Table 4). In these studies, the average age ranged from 23.6 to 35 years, average weight from 69 to 83 kg, CL from 11.2 to 19.7 L/h, and V_ss_ from 11.7 to 20.6 L. Therefore, our data, when combined with patient data, may serve as a robust baseline for building models with excellent predictive performance.

Li et al. compared the predictive performance of existing meropenem PK models and found that the 18 models were developed using critically ill patients, which exhibited a wide range of CL and V_ss_ values, from 2.18 to 16.7 L/h and 8.30 to 51.2 L, respectively [19]. Many of these studies involved patients on life-support, such as renal replacement therapy or ECMO, which contributed to the variability of the PK parameters. More significantly, the critical condition of these patients is often accompanied by various pathophysiological changes. These changes, whether the result of infection or noninfectious pathological conditions, can affect CL and V_ss_ [20]. For example, increased cardiac output can result in increased clearance, whereas third spacing altered protein binding, or both, can result in an increased volume of distribution. Similarly, renal and hepatic dysfunction may result in increased distribution and decreased clearance [20]. Gijsen et al. performed a population PK analysis focusing solely on ICU patients with normal or enhanced renal function, excluding those with an eGFR < 70 mL/min/1.73m^2^ as calculated by the CKD-EPI equation. CL and V_ss_ were estimated to be 13.7 L/h and 37.9 L, respectively [21]. These results indicate that in critically ill patients, V_ss_ may be significantly higher, whereas clearance rates are comparable to those in healthy individuals. The idea of augmented renal clearance (ARC) was proposed based on studies of patients who experienced subtherapeutic antimicrobial concentrations when administered standard doses. This suggests a criterion of a CL_CR_ of 130 mL/min/1.73 m^2^ or higher [22], which has since been broadly accepted based on follow-up studies and consensus [23]. Although numerous studies recommend increasing meropenem doses in patients with ARC [8,9,24,25,26], there have been studies establishing a prospective population pharmacokinetic model specifically for this cohort on meropenem or that provide dosing recommendations based on a PK model.

In the present study, Monte Carlo simulations were used to evaluate optimal dosing regimens for meropenem in adults with normal renal function. Beta-lactams, which are known for their time-dependent killing properties, have a PK/PD index in which the *f*T_>MIC_ correlates with therapeutic outcomes. Various in vitro studies, animal experiments, and clinical trials have demonstrated that for beta-lactams, both *f*T_>MIC_ and *f*T_>4MIC_ are related to rates of bactericidal effect, bacteriological eradication, or clinical cure [27,28,29]. For meropenem, the proposed ranges for *f*T_>MIC_ or *f*T_>4MIC_ vary from 40% to 100%. In the present study, 40%*f*T_>MIC_, 40%*f*T_>4MIC_, 100%*f*T_>MIC_, and 100%*f*T_>4MIC_ were designated as the efficacy targets to conduct the simulations [30,31,32,33,34,35]. In the present study, when pathogens had an MIC of 1, administering 2 g of meropenem every 6 h as a 3 h extended infusion only achieved a 30% PTA for 100%*f*T_>4MIC_ (Figure 3); however, a continuous infusion of 8 g surpassed a 90% PTA for 100%*f*T_>4MIC_ (Figure 4). This suggests that patients with normal or augmented renal function may require increased dosages or extended infusion times, which is consistent with several other studies. Hou et al. conducted simulations using pharmacokinetic parameters previously reported for patients with ARC [36] in which the clearance and volume of distribution were set at 19.2 ± 4.1 L/h and 25.35 ± 4.9 L, respectively. The results indicated that for a pathogen with an MIC of 1, administering 3 g every 6 h with an infusion time of either 0.5 or 3 h achieved a PTA of 86.6% for the 70%*f*T_>4MIC_ target [9]. Wang et al. also performed simulations using PK parameters derived from the NCA. They set the clearance (7.7 ± 1.8 L/h) and volume of distribution (22.6 ± 5.1 L) from a subgroup with normal CL_CR_. They observed that for targeting 100%*f*T_>4MIC_ with an MIC of 1 mg/L, only the regimen of 1.0 g every 6 h infused over 3 or 6 h achieved a PTA greater than 90% [10]. Furthermore, Sistanizad and colleagues reviewed antibiotic studies on ARC patients and recommended administering 2 g of meropenem every 6–8 h because prolonged infusion decreased the risk of subtherapeutic exposure [37].

There are several limitations to the present study. First, a variety of meaningful covariates cannot be detected with the small sample size of 12 participants. Despite testing various renal function indicators, such as CL_CR_ and eGFR, only serum creatinine levels showed a significant impact on CL in our model. The narrow range of renal function observed in our cohort of healthy adults may have further constrained the inclusion of other renal function indicators as meaningful covariates. However, this study is significant because it is the first to apply NCA and nonlinear mixed-effect modeling to evaluate the PK of meropenem in healthy Korean adults. In addition, it is expected to significantly assist with model development for our subsequent studies involving patients. Second, because the study was conducted in healthy individuals, it was not possible to assess the microbiological or clinical outcomes of meropenem treatment. Nevertheless, the simulations using PK parameters provided dosing recommendations that may be beneficial for patients. Third, the simulation results suggest dosages much higher compared with those typically used, indicating a need for validation in clinical studies.

In conclusion, this study provides important insights into the population PK of meropenem in healthy adults. It establishes a foundation for more accurate dosing guidelines tailored to patients with normal renal function. Through extensive Monte Carlo simulations, we demonstrated that current dosing strategies may require adjustment to optimize the therapeutic outcomes, particularly in patients with normal renal function. Our findings advocate for model-informed precision dosing regimens to ensure effective antimicrobial therapy, which will reduce the emergence of resistance and improve patient care.

## 4. Materials and Methods

### 4.1. Participants

This prospective study was conducted in January 2023 at the Clinical Trial Center of the Hallym University Sacred Heart Hospital. The selection of participants involved the following inclusion criteria:

(1) Adults between 19 and 55 years old at the time of screening.

(2) Individuals free from congenital, chronic conditions, and pathological signs during medical evaluations.

(3) Candidates considered eligible through comprehensive health assessments, which encompass medical histories, vital signs, physical exams, blood tests for hematology and biochemistry, urinalysis, and other diagnostic tests.

The exclusion criteria were:

(1) Participants with clinically significant conditions or histories of disorders in systems, such as gastrointestinal, cardiovascular, respiratory, endocrine, hepatobiliary, hematologic-oncologic, musculoskeletal, renal, neurological, psychiatric, immunological, urological, ophthalmological, otolaryngological, or genetic.

(2) Participants with past conditions known to influence the PK of medications, including liver or kidney impairment.

(3) Participants with known allergies to meropenem or previous adverse reactions to it.

(4) Participants testing positive for Hepatitis B surface antigen, hepatitis C virus antibodies, HIV antigen or antibodies, or syphilis.

(5) Women who are pregnant, breastfeeding, or may become pregnant.

### 4.2. Study Design

Study participants were administered 500 mg of meropenem intravenously over 30 min, which was prepared in 100 mL of saline solution. Venous blood (6 mL) was collected into tubes containing EDTA at specific time points to assess the PK characteristics. Sample collections occurred just before infusion (0 h), shortly after the dose (0.5 h), and at 0.75, 1, 2, 3, and 6 h after the infusion commenced. A validated high-performance liquid chromatography-tandem mass spectrometry assay was used to measure plasma levels of meropenem [38].

### 4.3. Population Pharmacokinetic Analysis

The PK parameters for meropenem were analyzed by nonlinear mixed-effects models using the NONMEM software package (version 7.5, ICON Clinical Research LLC, North Wales, PA, USA). Parameter estimation incorporated both fixed and random effects using the first-order conditional estimation with interaction method. This facilitated the modeling of interactions between interindividual variability (IIV) in PK parameters and residual variability (RV) in measured concentrations. The PK analysis of meropenem used one-, two-, and three-compartment models, which were established with the NONMEM PK model library subroutines ADVAN1 TRANS2, ADVAN3 TRANS4, and ADVAN11 TRANS4, respectively. Except for zero-order infusion processes, each model followed first-order kinetics. The models described each PK parameter using the equation θi = θ × exp(ηi), where θ represents the typical value, θi the individual parameter value, and ηi encapsulates the IIV, which was assumed to be normally distributed, having a mean of zero and a variance of ω^2^. RV was assumed to follow a normal distribution, having a mean of zero and a variance of σ^2^. Evaluations of RV considered additive, proportional, and combined additive–proportional error models. Model selection and evaluation were based on changes in NONMEM OFVs, parameter estimate precision indicated by relative standard errors, goodness-of-fit plots, VPC, and bootstrap analyses. Significant improvements in the model structures were evident when reductions in OFV exceeded thresholds of 3.84 (for one degree of freedom χ^2^ test) or 5.99 (for two degrees of freedom), indicating statistical significance at *p* < 0.05. Model validation involved generating plots of CWRES against time and population predictions (PREDs), and plots comparisons of actual data points against PREDs and individual predictions. VPC aligned the observed meropenem concentrations within 80% prediction intervals from 1000 model simulations. Variability in the predictions of the final model was analyzed using median and 95% confidence intervals from 2000 bootstrap samples.

Significant covariates affecting the PK parameters were identified through a stepwise forward selection and backward elimination process, with inclusion and exclusion thresholds set at *p* < 0.01 (ΔOFV < −6.635) and *p* < 0.001 (ΔOFV > 10.83), respectively. Covariate analysis included demographic (gender, age, weight, height, body mass index, BSA) and biochemical parameters (serum protein, albumin, creatinine, cystatin C levels), as well as the effect of renal clearance on meropenem elimination using the Cockcroft–Gault, MDRD, and CKD-EPI formulas. The Perl-speaks-NONMEM (PSN, version 5.3.1, https://uupharmacometrics.github.io/PsN/, accessed on 13 April 2023) tool was used for covariate identification, VPC execution, and nonparametric bootstrapping to evaluate model stability. Post-analysis processing and graphical representation of the outcomes were performed in the R programming environment (version 4.4.0, https://www.r-project.org/, accessed on 6 June 2024).

### 4.4. Noncompartmental Analysis

Noncompartmental analysis (NCA) was used to assess the plasma concentration-time profiles of meropenem and employed the R programming language and the NonCompart package. The evaluated PK parameters included maximum observed concentration (C_max_), time of last measurable concentration (T_last_), and concentration corresponding to T_last_ (C_last_). Additional parameters included the area under the concentration-time curve from the start of the dose to the last quantifiable concentration (AUC_last_) and from the start of dosing to infinity (AUC_inf_). The AUMC from time zero to T_last_ (AUMC_last_) and extrapolated to infinity (AUMC_inf_), mean residence time to infinity (MRT_inf_), total body clearance (CL_NCA_), volume of distribution as determined by NCA (V_zNCA_), steady state volume of distribution (V_ssNCA_), and terminal elimination half-life (t_1/2λz_) were also calculated. C_max_, T_last_, and C_last_ were directly determined from the observed data. The trapezoidal method with linear up and log down was used for the calculation of the AUC_last_ and AUMC_last_. AUC_inf_ was estimated by adding the ratio of C_last_ to the terminal elimination rate constant (λ_z_) to AUC_last_, which was obtained by log-linear regression of the terminal phase plasma concentrations. AUMC_inf_ was calculated using the formula AUMC_last_ + (T_last_ × C_last_)/λ_z_ + Clast/λ_z_^2^. The formula for MRT_inf_ was AUMC_inf_/AUC_inf_ minus half the infusion time. CL_NCA_ was determined as the dose divided by AUC_inf_, V_zNCA_ as CL_NCA_ divided by λ_z_, V_ssNCA_ as the product of MRT_inf_ and CL_NCA_, and t_1/2λz_ as the natural logarithm of two divided by λ_z_.

### 4.5. Dosage Simulation

To develop dosage recommendations for meropenem in patients with normal renal function, Monte Carlo simulations were carried out based on the final PK model. A total of 10,000 individual PK parameters for virtual patients were generated, assuming a log-normal distribution for each parameter or covariate. MIC values ranging from 0.06 to 16 mg/L were randomly assigned and reflected the distribution of meropenem MICs for *P. aeruginosa* as reported by the EUCAST. Among 59,460 cases, the distribution of MIC values was as follows: 0.06 mg/L for 4.43% (2634 cases), 0.125 mg/L for 10.14% (6028 cases), 0.25 mg/L for 18.29% (10,875 cases), 0.5 mg/L for 20.15% (11,982 cases), 1 mg/L for 14.81% (8807 cases), 2 mg/L for 8.68% (5161 cases), 4 mg/L for 6.35% (3776 cases), 8 mg/L for 5.46% (3247 cases), and 16 mg/L for 6.64% (3950 cases).

Steady state concentration-time profiles were generated for the virtual patients to assess the PTA. The therapeutic targets included 40%*f*T_>MIC_, 40%*f*T_>4MIC_, 100%*f*T_>MIC_, and 100%*f*T_>4MIC_, with a dosing regimen considered optimal if the PTA was 90% or higher. The fraction of unbound drug (*f*) was fixed at 0.98. An initial simulation assessed the adequacy of the current dosing regimen for patients with normal renal function, specifically for those with a CL_CR_ > 50 mL/min, using 1 g of meropenem administered every 8 h (q8h). This regimen was consistent with the recommended dosage for treating infections caused by *P. aeruginosa*, which also specifies a dose of 1 gram every 8 h. Subsequent simulations were conducted to establish meropenem dosage recommendations for patients with normal renal function. These analyses calculated the PTA for 10,000 patients at four dosing levels (0.6, 1, 1.5, and 2 g), two infusion durations (0.5 and 3 h), and three dosing intervals (6, 8, and 12 h). Further simulations were carried out to refine dosing recommendations for meropenem in continuous infusion settings for patients with normal renal function. This final simulation evaluated the PTA for extremely high dosages (e.g., 2, 4, 6, and 8 g), which were administered as continuous infusions. The analysis aimed to determine the PTA for achieving 100%*f*T_>MIC_ and 100%*f*T_>4MIC_.

## Figures and Tables

**Table 1:** Subject characteristics.

Parameters	Mean (CV%)	Median (IQR)
Demographic characteristics		
Age, years	36.8 (19.9%)	36.0 (31.5–39.3)
Height, cm	168 (4.29%)	168 (163–173)
Weight, kg	65.7 (20.8%)	61.7 (56.7–73.3)
Body surface area, m^2^	1.74 (11.6%)	1.71 (1.61–1.88)
Body mass index, kg/m^2^	23.0 (13.9%)	21.5 (21.4–24.0)
Laboratory characteristics		
Protein, g/dL	7.48 (4.64%)	7.45 (7.28–7.63)
Albumin, g/dL	4.88 (4.35%)	4.80 (4.78–5.03)
Cystatin C, mg/dL	0.790 (15.9%)	0.765 (0.705–0.873)
Creatinine, mg/dL	0.863 (19.0%)	0.860 (0.738–1.02)
Total bilirubin (mg/dL)	0.558 (62.2%)	0.470 (0.328–0.720)
Blood urea nitrogen, mg/dL	15.1 (30.0%)	14.1 (12.1–17.9)
Alanine aminotransferase, U/L	21.8 (85.3%)	17.0 (10.8–23.3)
Aspartate aminotransferase, U/L	25.0 (38.7%)	21.0 (19.8–27.5)
Gamma-glutamyl transferase, U/L	27.3 (68.1%)	17.0 (14.5–38.3)
Renal functions		
CL_CR_, CG (mL/min) ^a^	105 (21.2%)	105 (84.3–118)
CL_CR_, normalized CG (mL/min/1.73m^2^) ^b^	93.8 (16.4%)	93.2 (80.5–106)
eGFR, MDRD (mL/min/1.73m^2^) ^c^	108 (13.9%)	108 (96.6–116)
eGFR, CKD-EPI_CR_ (mL/min/1.73m^2^) ^d^	111 (15.4%)	110 (102–120)
eGFR, CKD-EPI_CC_ (mL/min/1.73m^2^) ^e^	112 (14.9%)	111 (102–122)
eGFR, CKD-EPI_CR-CC_ (mL/min/1.73m^2^) ^f^	93.3 (14.4%)	93.1 (82.9–98.8)
eGFR, adjusted MDRD (mL/min) ^g^	107 (11.5%)	111 (98.0–116)
eGFR, adjusted CKD-EPI_CR_ (mL/min) ^g^	110 (12.6%)	116 (102–121)
eGFR, adjusted CKD-EPI_CC_ (mL/min) ^g^	112 (12.3%)	116 (100–120)
eGFR, adjusted CKD-EPI_CR-CC_(mL/min/1.73m^2^) ^g^	101 (15.8%)	100 (90.7–112)

CV, coefficient of variation; IQR, interquartile range; CL_CR_, creatinine clearance; CG, Cockcroft–Gault equation; eGFR, estimated glomerular filtration rate; MDRD, modification of diet in renal disease; CKD-EPI, chronic kidney disease epidemiology collaboration; CR, creatinine; CC, cystatin C; min, the minimum of (CR or CC)/number and 1; max, the maximum of (CR or CC)/number and 1. ^a^ CLCR, CG = (140−Age) × weight/CR × 72 (× 0.85 if female). ^b^ CLCR, CG (normalized) = CLCR, CG / BSA × 1.73 m^2^. ^c^ eGFR = 175 × CR^−1.154^ × Age^−0.203^ (× 0.742 if female) ^d^ eGFR (female) = 142 × min (CR/0.7,1)^−0.241^ × max (CR/0.7,1)^−1.200^ × 0.9938^Age^ × 1.012 eGFR (male) = 142 × min (CR/0.9,1)^−0.302^ × max (CR/0.9,1)^−1.200^ × 0.9938^Age^. ^e^ eGFR (female) = 133 × min (CC/0.7,1)^−0.499^ × max (CC/0.7,1)^−1.328^ × 0.9962^Age^ × 0.932 [if female] ^f^ eGFR (female) = 135 × min (CR/0.7,1)^−0.219^ × max (CR/0.7,1)^−0.544^ × min (CC/0.8,1)^0.323^ × max (CC/0.8,1)^−0.778^ × 0.9961^Age^ × 0.963 eGFR (male) = 135 × min (CR/0.9,1)^−0.144^ × max (CR/0.9,1)^−0.544^ × min (CC/0.8,1)^0.323^ × max (CC/0.8,1)^−0.778^ × 0.9961^Age^. ^g^ The adjusted eGFR by MDRD and CKD-EPI equations are eGFR (adjusted) = eGFR (MDRD or CKD-EPI)/1.73 m^2^ × BSA.

**Table 2:** Parameter estimates and bootstrap medians (95% confidence intervals) for the final pharmacokinetic model of meropenem in 12 healthy adult participants.

Parameter	Estimate	RSE (%) [Shrinkage, %]	Bootstrap Median (95% CI)
Structural model			
CL = θ_1_ × (CR/0.86)^θ2^			
θ_1_ (L/h)	12.4	7.87	12.3 (10.8–14.7)
θ_2_	−0.392	19.2	−0.378 (−0.579–−0.115)
V1 (L)	8.26	12.5	8.31 (6.56–11.0)
Q (L/h)	5.22	16.1	5.05 (3.34–7.33)
V2 (L)	4.06	11.1	4.01 (3.00–5.07)
Interindividual variability			
CL (%)	26.2	30.4 [1.82]	25.4 (7.8–38.3)
V1 ^a^	1.53	4.80	1.53 (1.07–2.43)
Q (%) ^b^	14.4	[49.1]	
V2 (%) ^b^	17.9	[11.2]	
Residual variability			
Proportional error (%)	10.9	20.4	10.4 (6.50–14.0)

RSE, relative standard error; CI, confidence interval; CL, total clearance; V1, central volume of distribution; V2, volume of distribution for the first peripheral compartment; Q, intercompartmental clearance between V1 and V2; CR, serum creatinine level; a, the estimate indicates that the interindividual variability (IIV) of V1 is 1.53 times the IIV of CL; b, fixed.

**Table 3:** Descriptive statistics for the individual pharmacokinetic parameters from noncompartmental and population PK analysis.

Parameters	Unit	Mean (CV%)	Median (IQR)
NCA results			
C_max_	mg/L	40.2 (30.1%)	43.3 (34.2–46.3)
C_last_	h	0.393 (71.4%)	0.289 (0.231–0.484)
T_last_	mg/L	5.83 (7.56%)	6.00 (6.00–6.00)
AUC_last_	mg·h/L	39.8 (22.7%)	41.4 (35.9–44.9)
AUC_inf_	mg·h/L	40.4 (22.8%)	42.3 (36.2–45.2)
AUMC_last_	mg·h^2^/L	49.8 (22.1%)	51.0 (42.9–54.3)
AUMC_inf_	mg·h^2^/L	53.9 (24.1%)	54.2 (46.2–58.9)
MRT_inf_	h	1.09 (13.3%)	1.07 (0.989–1.22)
CL_NCA_	L/h/kg	0.201 (21.1%)	0.193 (0.174–0.218)
V_zZNCA_	L/kg	0.280 (25.7%)	0.260 (0.236–0.296)
V_ssNCA_	L/kg	0.219 (25.8%)	0.210 (0.187–0.228)
t_1/2λz_	h	0.967 (15.3%)	0.908 (0.870–1.08)
Population PK results			
CL	L/h/kg	0.197 (20.8%)	0.189 (0.169–0.215)
V_C_	L/kg	0.134 (37.7%)	0.118 (0.108–0.137)
V_ss_	L/kg	0.197 (25.4%)	0.184 (0.165–0.209)
AUC	mg·h/L	41.3 (21.7%)	43.5 (37.1–45.2)
t_1/2α_	h	0.260 (26.9%)	0.229 (0.214–0.277)
t_1/2β_	h	0.985 (14.5%)	0.923 (0.893–1.10)

CV, coefficient of variation; IQR, interquartile range; C_max_, maximum observed plasma concentration; T_last_, time of last measurable concentration; C_last_, concentration corresponding to T_last_; AUC_last_, area under the plasma concentration-time curve (AUC) from the start of the dose to the last quantifiable concentration; AUC_inf_, AUC from the start of dosing to infinity; AUMC_last_, area under the first moment curve (AUMC) from 0 h to the T_last_; AUMC_inf_, AUMC extrapolated to infinity, based on the last observed concentration; MRT_inf_, mean residence time from 0 h to infinite; CL_NCA_, total body clearance determined by NCA; V_zNCA_, volume of distribution (Vd) determined by NCA; V_ssNCA_, steady state Vd determined by NCA; _t1/2λz_, terminal elimination half-life. Formulas: AUC_inf_, AUC_last_ + C_last_/λ_z_; AUMC_inf_, AUMC_last_ + (T_last_ × C_last_)/λ_z_ + Clast/λ_z_^2^; MRT_inf_, AUMC_inf_/AUC_inf_ − infusion time/2; CL_NCA_, dose/AUC_inf_; V_zNCA_, CL_NCA_/λ_z_; V_ssNCA_, MRT_inf_ × CL_NCA_; t_1/2λz_, ln (2)/λ_z_; CL, total clearance; V_C_, central volume of distribution; V_ss_, steady state volume of distribution; AUC, dose/CL; t_1/2α_, distribution phase half-life; t_1/2β_, elimination phase half-life

**Table 4:** Summary of the demographic characteristics and pharmacokinetic properties of meropenem in healthy participants.

Study	n	Age (Years)	Weight (kg)	Height (cm)	BSA (m^2^)	CL (L/h)	V_ss_ (L)
Bax et al. [12]	12	26 (19–45)	74 (68–87)	179 (170–184)	1.92 *	16.7 (1.2)	19.1 (1.6)
Wise et al. [13]	6	23.6 (23–31)	69.9 (63–80)	180 (169–187)	1.89 *	15.2 (3.09)	20.6 (5.90)
Burman et al. [14]	6	35 (30–40)	83 (68–93)	–	–	16.6 (0.6)	20.4 (0.7)
Nilsson-Ehle et al. [15]	8	33 (22–38)	74 (66–86)	–	–	11.3 (1.86)	12.5 (1.50)
Christensson et al. [16]	6	34 (13.4)	79 (8.4)	–	1.96 (0.09)	11.2 (1.68)	14.7 (0.21)
Leroy et al. [17]	6	33.8 (9.0)	66.9 (12.4)	–	–	19.7 (5.7)	27.4 (7.0)
Ljunberg et al. [18]	8	28 (5.2)	69 (7.7)	–	1.88 (7.3)	11.7 (1.7)	11.7 (1.2)

The values are mean (range or SD). BSA, body surface area; CL, total clearance; V_ss_, total volume of distribution at steady state; *, calculated based on the average body weight and height using the de Bois method. (BSA = 0.007185 × height^0.725^ × weight^0.425^).

## Data Availability

The datasets generated and/or analyzed during the current study are available from the corresponding author upon reasonable request.

## Supplementary Materials

The following supporting information can be downloaded at: https://www.mdpi.com/article/10.3390/antibiotics13090849/s1, Figure S1. Individual fit plots for meropenem (a) normal scale, (b) semi-log scale: closed circles, observed concentrations; solid line, individual predicted concentrations; dotted line, population predicted concentrations. Figure S2. Visual predictive check from simulated concentrations of 1,000 virtual datasets of meropenem: closed circles, observed concentrations; solid lines, 10th, 50th, and 90th percentiles of observations; dashed lines, 10th, 50th, and 90th percentiles of simulated concentrations and shaded areas, 95% confidence intervals for the 10th, 50th, and 90th percentiles of the simulated concentrations. Table S1. Parameter estimates for the base final pharmacokinetic model of meropenem in 12 healthy adult participants. Table S2. Stepwise covariate selection process: forward selection (*p*-value = 0.01, OFV difference < −6.635, degree of freedom = 1) and backward elimination (*p*-value = 0.001, OFV difference < 10.83, degree of freedom = 1).
