# Supplementary material for: Precision Dosing of Meropenem in Adults with Normal Renal Function: Insights from a Population Pharmacokinetic and Monte Carlo Simulation Study
Source: Antibiotics (Basel). 2024 Sep 5;13(9):849. doi: 10.3390/antibiotics13090849 (PMC11429322; doi:10.3390/antibiotics13090849)
Supplement: Supplementary file 1 [file antibiotics-13-00849-s001.zip › antibiotics-3164152-supplementary.pdf]

Supplementary Figures

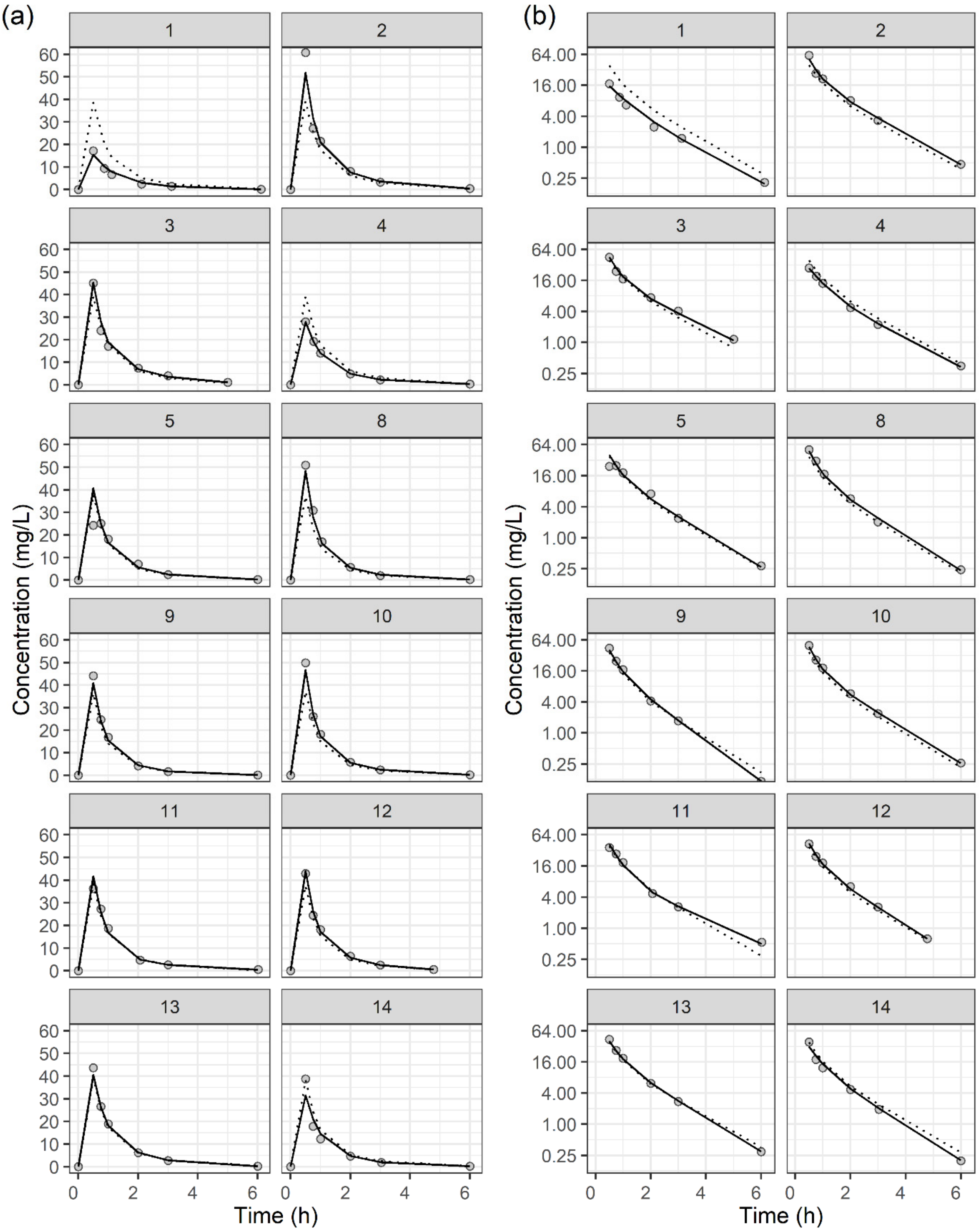

Figure S1. Individual fit plots for meropenem (a) normal scale, (b) semi-log scale: closed circles, observed concentrations; solid line, individual predicted concentrations; dotted line, population predicted concentrations.

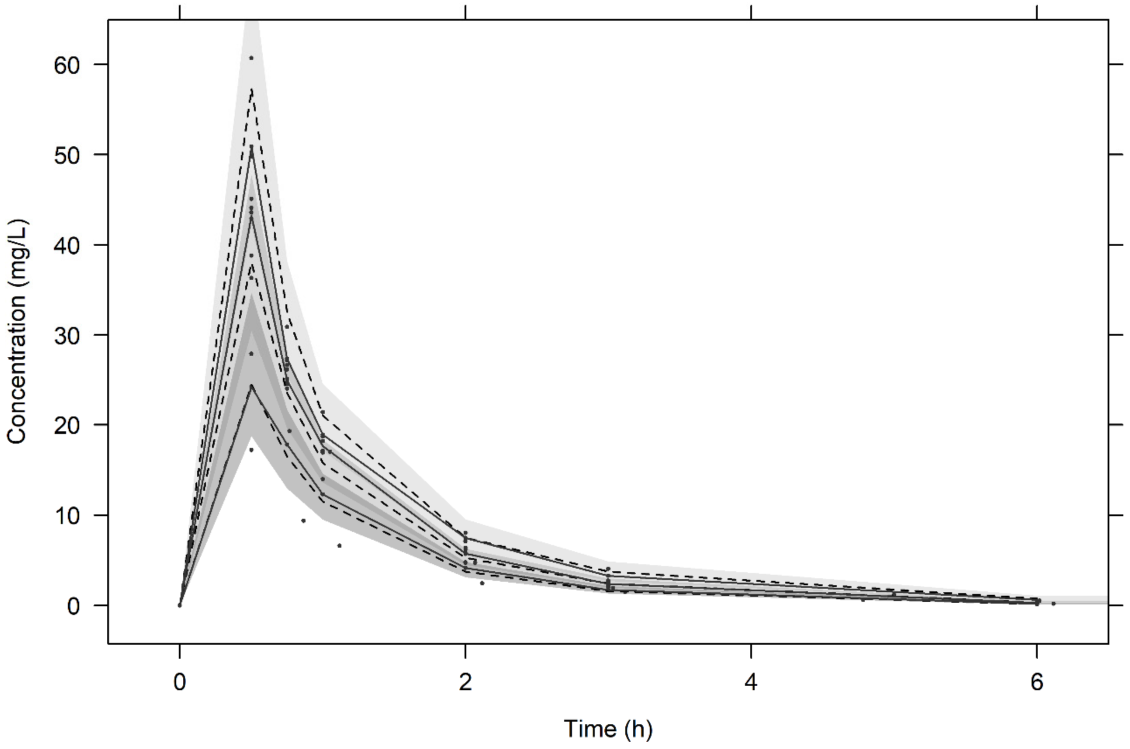

Figure S2. Visual predictive check from simulated concentrations of 1,000 virtual datasets of meropenem: closed circles, observed concentrations; solid lines, 10th, 50th, and 90th percentiles of observations; dashed lines, 10th, 50th, and 90th percentiles of simulated concentrations and shaded areas, 95% confidence intervals for the 10th, 50th, and 90th percentiles of the simulated concentrations.

Table S1. Parameter estimates for the base pharmacokinetic model of meropenem in 12 healthy adult participants

Parameter	Estimates	RSE(%) [Shringkage, %]
Structural model		
$CL = \theta_1 \times (CR / 0.86)^{\theta 2}$		
θ_1 (L/h)	12.4	7.56
V1	7.86	13.6
Q	5.83	15.4
V2	4.31	9.88
Interindividual variability		
CL (%)	25.4	31.1 [2.24]
V1 [a]	1.59	4.4
Q (%) [b]	21.4	[36.0]
V2 (%) [b]	25.9	[6.30]
Residual variability		
Proportional error (%)	11.3	16.2

RSE, relative standard error; CL, total clearance; V1, central volume of distribution; V2, volume of distribution for the first peripheral compartment; Q, intercompartmental clearance between V1 and V2; CR, serum creatinine level; a, The estimate indicates that the interindividual variability (IIV) of V1 is 1.59 times the IIV of CL; b, Fixed.

Table S2. Stepwise covariate selection process: forward selection (p-value = 0.01, OFV difference < -6.635, degree of freedom = 1) and backward elimination (p-value = 0.001, OFV difference < 10.83, degree of freedom = 1)

Parameter	Covariate	Base OFV	New OFV	OFV difference	P-value
Forward step 1					
CL	Albumin	-160.43648	-167.71807	-7.28159	0.006966
CL	Cystatin C	-160.43648	-172.93916	-12.50268	0.000406
CL	Creatinine	-160.43648	-175.61037	**-15.17389**	0.000098
CL	CL_{CR}, CG [a]	-160.43648	-168.30816	-7.87168	0.005021
CL	eGFR, MDRD [b]	-160.43648	-172.65232	-12.21584	0.000474
CL	eGFR, CKD-EPI_{CR} [c]	-160.43648	-174.98057	-14.54409	0.000137
CL	eGFR, CKD-EPI_{CC} [d]	-160.43648	-170.46016	-10.02368	0.001545
CL	eGFR, CKD-$EPI_{CR\text{-}CC}$ [e]	-160.43648	-173.14571	-12.70923	0.000364
CL	Sex	-160.43648	-172.94164	-12.50516	0.000406
V1	Sex	-160.43648	-175.51332	-15.07684	0.000103
V2	Sex	-160.43648	-170.14345	-9.70697	0.001836
V2	Weight	-160.43648	-167.08191	-6.64543	0.009941
Forward step 2					
CL	Albumin	-175.61037	-184.55277	**-8.94239**	0.002786
CL	Protein	-175.61037	-182.64174	-7.03137	0.008009
V1	Albumin	-175.61037	-183.74746	-8.13709	0.004337
V1	Protein	-175.61037	-182.59633	-6.98596	0.008215
Forward step 3					
V2	Age	-184.55277	-191.78634	-7.23358	0.007155
V2	Body mass index	-184.55277	-192.64283	**-8.09006**	0.004451
Forward step 4					
Nothing					
Backward step 1					
V2	Body mass index	-192.64283	-184.55277	**8.09006**	0.004451
Backward step 2					
CL	Albumin	-184.55277	-175.61037	**8.9424**	0.002786
Backward step 3					
Nothing					

OFV, objective function value; CL_{CR}, creatinine clearance; CG, Cockcroft-Gault equation; eGFR, estimated glomerular filtration rate; MDRD, modification of diet in renal disease; CKD-EPI, chronic kidney disease epidemiology collaboration; CR, creatinine; CC, cystatin C; min, the minimum of (CR or CC)/number and 1; max, the maximum of (CR or CC)/number and 1

[a] CL_{CR}, CG = (140−Age) × weight/CR × 72 (× 0.85 if female)

[b] eGFR = 175 × $CR^{-1.154}$ × $Age^{-0.203}$ (× 0.742 if female)

[c] eGFR (female) = 142 × min $(CR/0.7,1)^{-0.241}$ × max $(CR/0.7,1)^{-1.200}$ × 0.9938^{Age} × 1.012

eGFR (male) = 142 × min $(CR/0.9,1)^{-0.302}$ × max $(CR/0.9,1)^{-1.200}$ × 0.9938^{Age}

[d] eGFR (female) = $133 \times \min(CC/0.7,1)^{-0.499} \times \max(CC/0.7,1)^{-1.328} \times 0.9962^{Age} \times 0.932$ [if female]

[e] eGFR (female) = $135 \times \min(CR/0.7,1)^{-0.219} \times \max(CR/0.7,1)^{-0.544} \times \min(CC/0.8,1)^{0.323} \times \max(CC/0.8,1)^{-0.778} \times 0.9961^{Age} \times 0.963$

eGFR (male) = $135 \times \min(CR/0.9,1)^{-0.144} \times \max(CR/0.9,1)^{-0.544} \times \min(CC/0.8,1)^{0.323} \times \max(CC/0.8,1)^{-0.778} \times 0.9961^{Age}$
